# The Missing Medians: Exclusion of Ordinal Data from Meta-Analyses

**DOI:** 10.1371/journal.pone.0145580

**Published:** 2015-12-23

**Authors:** Toby B. Cumming, Leonid Churilov, Emily S. Sena

**Affiliations:** 1 Florey Institute of Neuroscience and Mental Health, University of Melbourne, Melbourne, Australia; 2 Centre for Clinical Brain Sciences, School of Clinical Sciences, University of Edinburgh, Edinburgh, United Kingdom; Universitat Wien, AUSTRIA

## Abstract

**Background:**

Meta-analyses are considered the gold standard of evidence-based health care, and are used to guide clinical decisions and health policy. A major limitation of current meta-analysis techniques is their inability to pool ordinal data. Our objectives were to determine the extent of this problem in the context of neurological rating scales and to provide a solution.

**Methods:**

Using an existing database of clinical trials of oral neuroprotective therapies, we identified the 6 most commonly used clinical rating scales and recorded how data from these scales were reported and analysed. We then identified systematic reviews of studies that used these scales (via the Cochrane database) and recorded the meta-analytic techniques used. Finally, we identified a statistical technique for calculating a common language effect size measure for ordinal data.

**Results:**

We identified 103 studies, with 128 instances of the 6 clinical scales being reported. The majority– 80%–reported means alone for central tendency, with only 13% reporting medians. In analysis, 40% of studies used parametric statistics alone, 34% of studies employed non-parametric analysis, and 26% did not include or specify analysis. Of the 60 systematic reviews identified that included meta-analysis, 88% used mean difference and 22% employed difference in proportions; none included rank-based analysis. We propose the use of a rank-based generalised odds ratio (WMW GenOR) as an assumption-free effect size measure that is easy to compute and can be readily combined in meta-analysis.

**Conclusion:**

There is wide scope for improvement in the reporting and analysis of ordinal data in the literature. We hope that adoption of the WMW GenOR will have the dual effect of improving the reporting of data in individual studies while also increasing the inclusivity (and therefore validity) of meta-analyses.

## Introduction

Combining results from multiple studies to more powerfully estimate effect size can be very informative. Meta-analysis, however, has several well documented weaknesses: bias in the original studies will flow through to the pooled data, and non-publication of negative studies (the ‘file drawer’ problem [1]) can lead to over-estimation of effect. Less well documented is the omission of ordinal data from meta-analyses (the ‘missing medians’ problem). In reporting study-specific summary statistics, many authors present means and standard deviations on the assumption that their data are continuous and normally distributed. Deriving effect sizes from such studies and pooling them in meta-analysis is straightforward [2]. In many cases, however, outcome measures are ordinal rather than continuous; a scale’s categories have a natural order, but it cannot be assumed that differences between the categories are equivalent. This is particularly common in clinical research, where scales are designed to evaluate impairment and be clinically meaningful. One example is in the stroke literature, where the modified Rankin Scale is the primary outcome of choice in the vast majority of trials. It is a measure of functional disability, and has a 7-point ordinal scale ranging from 0 (no symptoms) to 6 (dead). By generating disability weights from WHO Global Burden of Disease data, Hong & Saver [3] demonstrated empirically that the points on the scale are not equally spaced. Yet prominent recent stroke trials have summarised ordinal modified Rankin Scale data using means [4]. Additionally, many clinical scales suffer from ceiling or floor effects, yielding data that are not normally distributed. Interpreting means and standard deviations in these conditions is problematic; medians and inter-quartile ranges are statistically more valid.

These reporting considerations have important implications for meta-analysis. Where ordinal data are reported appropriately in individual studies, they are often excluded from meta-analysis due to the difficulty in pooling them. Alternatively, where study authors report means and standard deviations, often inappropriately, these data can be included in meta-analysis but the validity of the pooled results is questionable. Meta-analytical results are heavily influenced by treatment of outliers and by parametric versus non-parametric estimation [5]. The Cochrane collaboration acknowledge the problem with meta-analysis of ordinal or non-parametric data in their handbook (“difficulties will be encountered if studies have summarised their results using medians”, section 9.2.4[2]), but do not propose a solution. In practice, investigators often dichotomise data from shorter ordinal scales, and treat data from longer ordinal scales as continuous. Both of these approaches are sub-optimal. Dichotomising scales necessitates a loss of detail, and participants close to but on opposite sides of the split are characterised as very different rather than very similar. Statistical power is lost: a median split has been equated to discarding one-third of the data [6]. Treating data as continuous implies a consistent relationship between each level of the scale, which is not true of ordinal scales, and assumptions of normality are often violated. In the context of meta-analysis, it may be argued that, due to central limit theorem, mean values across a group of studies (and hence mean differences) will be approximately normally distributed, rendering any concerns about violation of normality invalid. Although this may be true, it fails to acknowledge that it is inappropriate to use means as a measure of central tendency for scales where we know only the order of levels on the scale, and not the distance between them.

Attempts to address these limitations were made more than 30 years ago. Kraemer and Andrews published a paper entitled “A non-parametric technique for effect size calculation” in 1982 [7]. This was followed in 1983 by a technical report from Hedges and Olkin entitled “Nonparametric estimators of effect size in meta-analysis” [8]. Yet there remains little agreement on how to pool ordinal data appropriately in meta-analysis. Some have suggested using a proportional odds model for combining data from ordinal scales [9,10], although this assumes a consistent relationship between levels on the scale. Others have developed techniques to estimate mean and standard deviation from the median and range [11,12], but this is an inexact solution, with estimates affected by sample size and normality of the data. Data may be pooled based on the Fisher’s exact test rather than the t-statistic [13], though this requires dichotomisation. A Bayesian approach that is not reliant on an assumption of normality has been proposed [14], but this necessitates complex modelling of the data.

## Methods

First, to identify the most widely used clinical rating scales, we analysed all the studies included in a systematic review of clinical trials of oral neuroprotective therapies in multiple sclerosis, Alzheimer’s disease, amyotrophic-lateral sclerosis, Parkinson’s disease, and Huntington’s disease [15]. These studies provide an insight into the use of neurological rating scales; they may not be representative of all conditions. We selected the 6 most common rating scales, and extracted data from each of the individual studies that included these scales on (a) the measure of central tendency reported and (b) the type of analysis used. Second, to assess how these data are pooled in meta-analysis, we searched the Cochrane Database (in September 2013) for systematic reviews using each of the 6 rating scales as a keyword. For each of the relevant systematic reviews, we extracted data on the meta-analytic approach used. Third, we identified an effect size measure for use in meta-analysis of ordinal data that is assumption-free and easy to compute. To demonstrate feasibility, we generated mock data and used this effect size measure in meta-analysis.

## Results and Discussion

### Reporting and analysis of individual study data

The 6 most commonly used rating scales were: the Mini-Mental State Examination (MMSE), the Expanded Disability Status Scale (EDSS), the Unified Parkinson’s Disease Rating Scale (UPDRS), the Alzheimer’s Disease Assessment Scale (ADAS), the Unified Huntington’s Disease Rating Scale (UHDRS) and the Amyotrophic Lateral Sclerosis Functional Rating Scale (ALSFRS). Properties of these 6 scales are outlined in the boxed text (Box 1).

**Box 1. Clinical Rating Scales.**

**MMSE**: Cognitive screening tool scored out of 30 [16], often treated as a continuous scale. Data are typically skewed towards ceiling, as illustrated in a post-stroke population (median 26, IQR 22–27, skewness -1.09) [17].

**EDSS**: Ordinal rating scale widely used to evaluate function in multiple sclerosis [18]. Ranges from 0–10 in half-point increments. Distribution of EDSS scores is rarely normal, with the predominant pattern being bimodal [19].

**UPDRS**: Ordinal rating scale widely used to assess function in Parkinson’s disease [20]. Includes 42 items that are mostly scored on a 5 point scale (0 normal, 4 most severe).

**ADAS**: Designed to screen for early Alzheimer’s disease. Includes a cognitive subscale (ADAS-cog) [21]. ADAS-cog is scored from 0–70 and is generally treated as a continuous scale.

**UHDRS**: Ordinal rating scale used to test function in Huntington’s disease [22]. Each of its 6 components has a different scoring format; they are typically reported separately.

**ALSFRS**: Ordinal rating scale used to measure function in amyotrophic lateral sclerosis. Revised to ALSFRS-R [23]. Contains 12 items, scored from 0 (most severe) to 4 (normal). Data are often skewed, with >80% of patients classed as ‘mild’ or ‘moderate’ (>24) [24].

**Fig 1:**
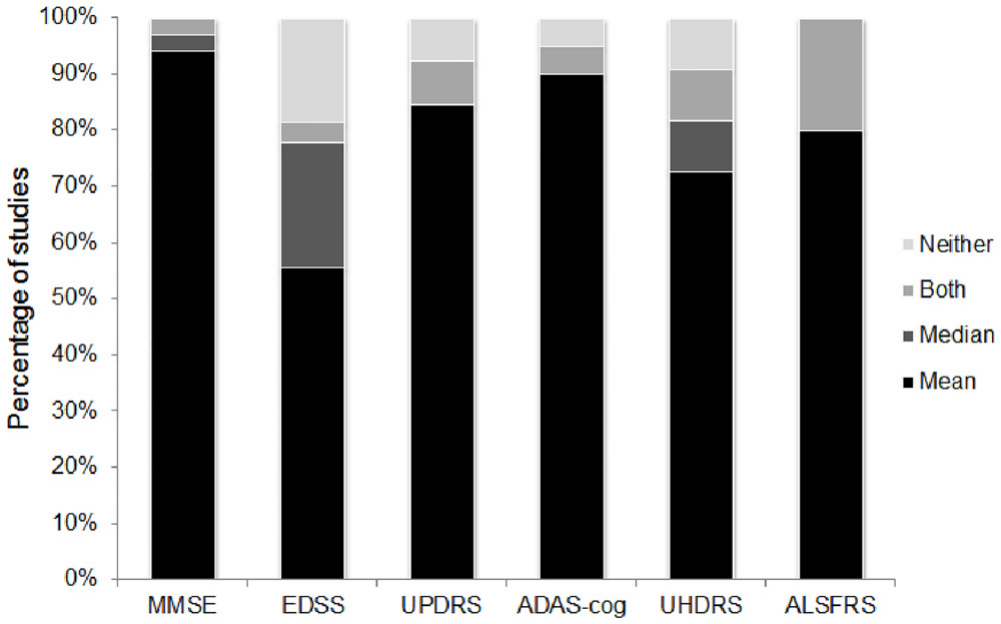
Reporting of central tendency for the 6 clinical rating scales.

Across 103 studies, we identified 128 instances of scale data being reported (MMSE 34, EDSS 27, UPDRS 26, ADAS-cog 20, UHDRS 11, ALSFRS 10). Of these, 7% did not include a measure of central tendency, 13% reported medians (either alone or alongside means) and 80% reported means alone (Fig 1).

**Fig 2:**
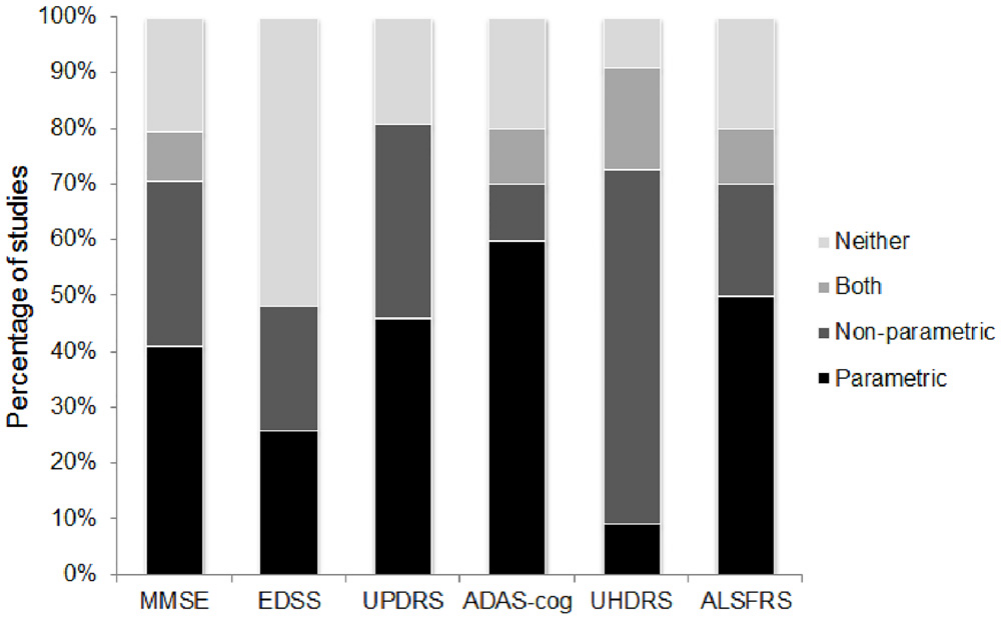
Approach to statistical analysis for the 6 clinical rating scales.

Of the 128 instances of scale data being reported, 26% did not include analysis or did not specify analysis type, 34% featured non-parametric (rank-based) analysis—either alone or alongside other analysis, and 40% included parametric (reliant on assumption of normality) analysis alone (Fig 2).

### Meta-analysis of rating scale data

**Fig 3:**
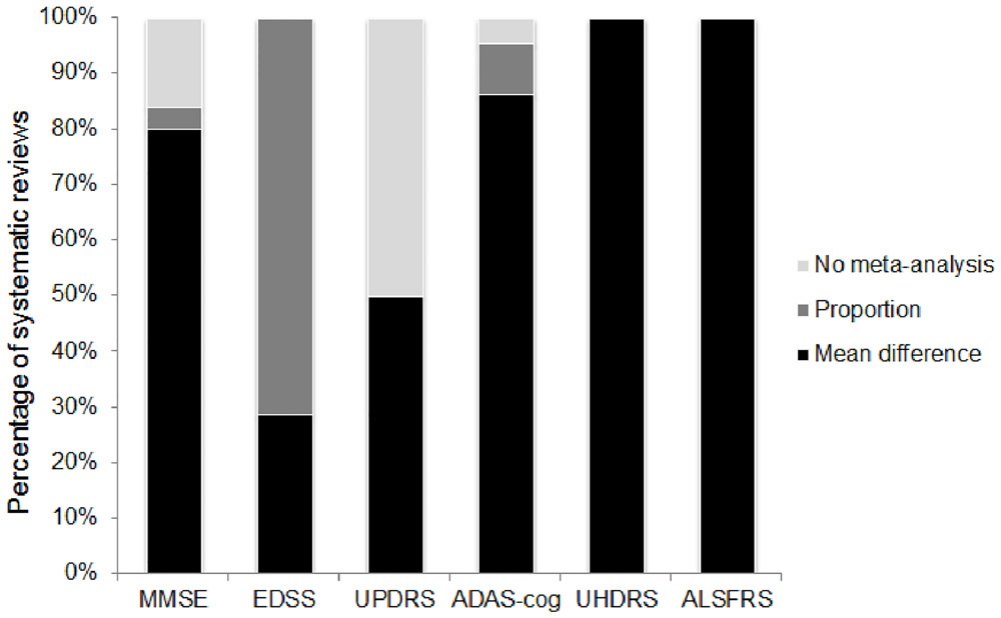
Approach to pooling data from the 6 clinical rating scales in systematic reviews.

We identified 70 relevant systematic reviews (MMSE 24, EDSS 11, UPDRS 10, ADAS-cog 20, UHDRS 1, ALSFRS 4), 60 of which incorporated meta-analysis. Of these 60, 88% included mean difference and 22% included difference in proportions (Fig 3). None included medians or other rank-based effect size measures in their meta-analyses. In fact, across all the systematic reviews, only a single study was identified that reported median difference as an outcome (on the UPDRS) [25].

### Why the status quo is a problem

In 4 out of every 5 studies, means were the single measure of central tendency reported, while only approximately 1 in every 8 studies included medians. Approach to data analysis revealed a mis-match; in studies where analyses were specified, almost half (46%) included a non-parametric technique. Acknowledgement that the data may not conform to parametric assumptions was stronger at the analysis stage than at the descriptive stage. Reliance on means, standard deviations and standardised mean differences in the context of ordinal assessment scales that often yield non-normally distributed data is likely to violate statistical assumptions and bias interpretation of results. Yet correct reporting of medians and inter-quartile ranges will disqualify the data from subsequent meta-analysis, not only reducing the available information but potentially selecting out higher quality studies. What is required is a method for reporting ordinal data that does not assume a certain underlying distribution, does not necessitate dichotomisation, that facilitates pooling of results in meta-analysis, and does not inflict too harsh a reporting burden on individual study authors.

### A simple solution: The generalised odds ratio

In pursuit of our third aim, we propose the use of a rank-based generalised odds ratio as a non-parametric equivalent to the standardised mean difference for use in meta-analysis. Agresti [26] outlined a generalisation of the odds ratio (GenOR) for ordinal outcomes. The method involves considering all possible pairs of observations, one from the treatment group and one from the control group. Agresti’s GenOR measures the ratio of the probabilities that a randomly chosen pair favours the treatment or control group. For example, if GenOR = 2, the clinical interpretation is that “compared to a person who does not receive the treatment, you are twice as likely to have a better outcome as you are to have a worse outcome.” Agresti’s GenOR measure, however, discards data where observations are tied. This can involve discarding a considerable amount of data, thus reducing precision in the estimate of treatment effect, and potentially biasing clinical interpretation. If GenOR = 2 and 50% of the observations are tied, the above interpretation still applies but is made more accurate with the addition of “…but there's also a 50% chance you will have the same outcome.”

The proposed Wilcoxon-Mann-Whitney generalised odds ratio (WMW GenOR) follows the same logic as Agresti’s GenOR but does not ignore the ties; tied observations are split evenly between better and worse outcomes [27]. We have published full details on how to calculate the WMW GenOR elsewhere [28] (S2 File). For a 2-group comparison, the WMW GenOR can be used as a natural effect size measure to accompany the WMW test. Importantly, the WMW GenOR can also be calculated for other outcomes, including continuous ones [27]. Interpretation is straightforward, as the odds-based measure can be directly translated to the ‘common language effect size’ measure [29]. Assuming normally distributed data with similar variances, this common language effect size can then be translated to the standardized mean difference. For example, for a certain fixed sample size, if the probability that a randomly selected person from the treatment group will score higher than a randomly selected person from the control group is 0.58, this equates to a standardized mean difference of 0.3; a probability of 0.71 equates to a standardized mean difference of 0.8 [30]. The WMW GenOR also has a natural relationship to the Number-Needed-to-Treat calculated on an ordinal scale [31]. Confidence intervals and p-values for WMW GenOR are implementable in any statistical software, including standard spreadsheets, given the existence of conveniently computable closed-form asymptotic expressions. Importantly, for the analysis of research trials where adjustment for prognostic covariates is often recommended, these formulas easily extend to stratified analyses.

Where individual studies have computed the WMW GenOR, the ln(GenOR) and the standard error can be inserted directly into meta-analysis (e.g., using *metan* command in Stata). While the sampling distribution of the odds ratio may be skewed, the sampling distribution of the log odds ratio follows an approximately normal distribution [26]. Where individual studies have not computed the WMW GenOR, it remains possible to calculate it retrospectively and include the data in meta-analysis. This requires the following information: sample size of the groups (N1, N2), Mann-Whitney U statistic and 2-tailed p-value. Ln(GenOR) is calculated as Ln[(U/(N1*N2))/(1-(U/(N1*N2))] and standard error as *ABS[Ln(GenOR)/Invnormal(1-p/2)]*. Although the WMW GenOR is different to an odds ratio generated from a study with a binary outcome, it can be meta-analysed and visually represented in forest plots in the same way.

### Implementation: An example using mock EDSS data

**Fig 4:**
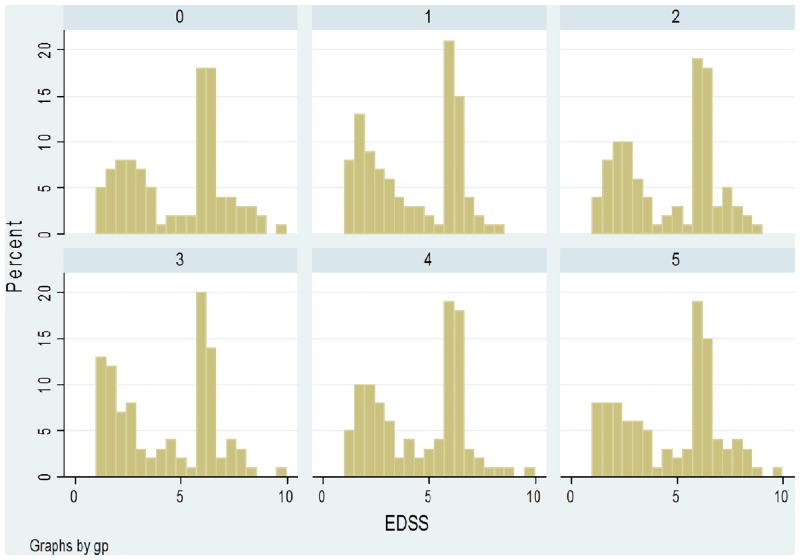
Histograms of mock EDSS data in a control group (sample 0) and 5 treatment groups (samples 1–5).

Using the basic distribution of EDSS scores identified in a large multiple sclerosis study [19] as a starting point, we generated mock data for a control group (sample 0) and 5 treatment groups (samples 1–5). Each group consisted of 100 patients. Shapiro-Wilk tests indicated that distribution of data in all samples was significantly non-normal. The histograms of EDSS scores are presented in Fig 4.

**Fig 5:**
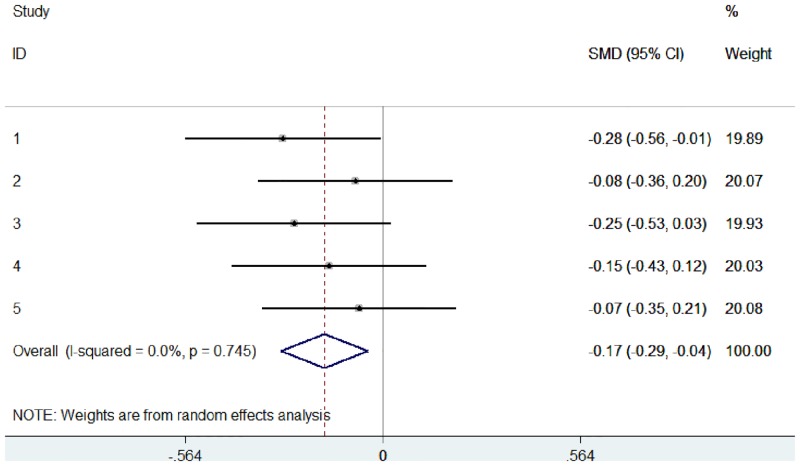
Random effects meta-analysis, based on standardised mean difference, of 5 simulated studies using mock EDSS data.

**Fig 6:**
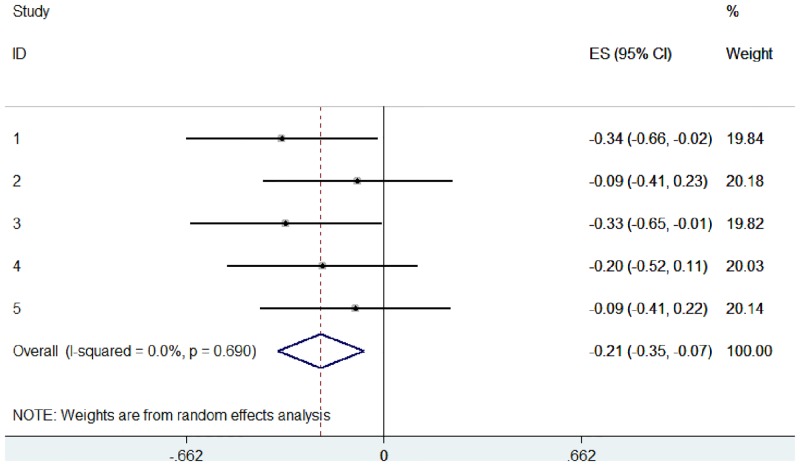
Ordinal meta-analysis, based on WMW GenOR, of 5 simulated studies using mock EDSS data.

These data were used to simulate 5 studies, with EDSS scores compared between control and treatment groups (Study 1: sample 0 versus sample 1, Study 2: sample 0 versus sample 2, etc.). Using these raw data, we pooled the 5 studies in 2 different ways: (1) a typical random effects meta-analysis based on the standardised mean difference (Fig 5), and (2) an ordinal meta-analysis based on the WMW GenOR (Fig 6).

**Fig 7:**
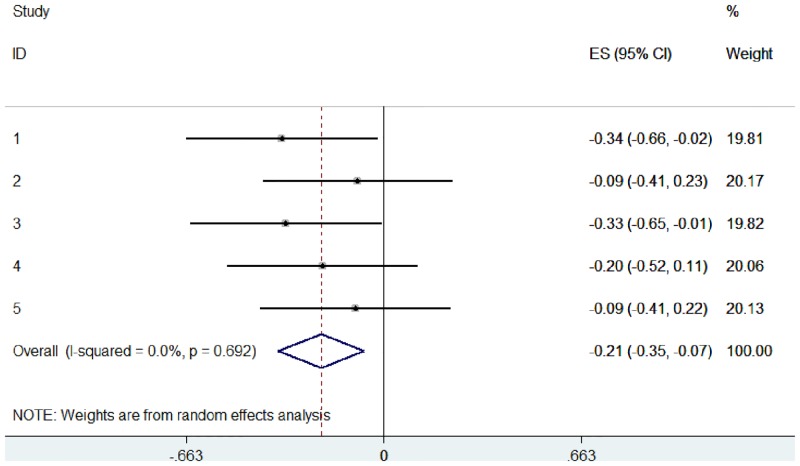
Ordinal meta-analysis, based on WMW GenOR calculated from summary statistics, of 5 simulated studies using mock EDSS data.

In practice, the WMW GenOR is rarely calculated at the study level and so this information is unavailable to authors of systematic reviews. To provide a more realistic implementation, we used the same mock data but assumed that only summary statistics were available from the 5 ‘studies’ (Table 1). These Mann-Whitney U and p-values were used to calculate WMW GenORs, which were then combined in ordinal meta-analysis (Fig 7). Calculating WMW GenORs retrospectively from summary data made no difference to effect sizes (compare Figs 6 and 7), indicating that meta-analysis is feasible even when WMW GenORs are not reported at the study level.

**Table 1:** Mock EDSS data: Summary statistics from the 5 ‘studies’.

Sample	N	Median (IQR)	Mann-Whitney U (vs sample 0)
0	100	6.0 (2.5, 6.5)	
1	100	4.25 (2.0, 6.0)	4155, z = -2.08 (p = .038)
2	100	6.0 (2.5, 6.5)	4782, z = -0.54 (p = .592)
3	100	4.5 (1.625, 6.375)	4186, z = -2.00 (p = .045)
4	100	5.25 (2.125, 6.5)	4490, z = -1.26 (p = .209)
5	100	5.75 (2.5, 6.5)	4764, z = -0.58 (p = .562)

These forest plots show that ordinal data can be visually represented and interpreted using meta-analysis in the same way as continuous or dichotomous data. WMW GenOR-based meta-analysis is not only the more appropriate approach for ordinal data, in this case it was more sensitive to group differences than the standardised mean difference approach (compare Figs 5 and 6). In Study 3 (sample 0 versus sample 3), group difference was significant according to Mann-Whitney U (p = 0.045) but not according to t-test (p = 0.073). It is often assumed that parametric analyses are more likely to detect significant treatment effects, but this is not necessarily true in the context of non-normal distributions.

## Conclusions

Meta-analysis is a vital tool for research and clinical decision-making. At the individual study level, it is important that ordinal data can be reported appropriately and not be excluded from meta-analysis. Using means and standard deviations in the context of ordinal scales gives an impression of exactness, but this is false precision; we do not know the distance between points on the scale, only their order. Our results demonstrate that there is wide scope for improvement in the reporting and analysis of ordinal and non-parametric data in the literature. A solution to this problem is the WMW GenOR. This odds-based effect size measure is assumption-free, easy to compute and can be readily combined in meta-analysis. Such a probability-based effect size is not limited to ordinal data; it is easily applied to continuous data too. We hope that adoption of the WMW GenOR will have the dual effect of improving the reporting of ordinal data in individual studies while also increasing the inclusivity (and therefore validity) of meta-analyses.

## Supporting Information

S1 File Excel file containing study data. (XLSX)

S2 File PDF file of related article (Churilov et al., 2014). (PDF)
