# Supplementary material for: The Missing Medians: Exclusion of Ordinal Data from Meta-Analyses
Source: PLoS One. 2015 Dec 23;10(12):e0145580. doi: 10.1371/journal.pone.0145580 (PMC4689383; doi:10.1371/journal.pone.0145580)
Supplement: S2 File — (PDF) [file pone.0145580.s002.pdf]

An improved method for simple, assumption-free ordinal analysis of the modified Rankin Scale using generalized odds ratios

Leonid Churilov[1,2], Sarah Arnup[1,3], Hayden Johns[1,2], Tiffany Leung[1,2], Stuart Roberts[1,2], Bruce C. V. Campbell[4], Stephen M. Davis[4], and Geoffrey A. Donnan[1]

Background and Purpose: **Developing improved methods for analysis of the modified Rankin Scale (mRS) remains a critical issue for the stroke research community. A recently proposed permutation-based approach is assumption-free and easily interpretable but computationally intensive and does not provide confidence intervals to quantify the precision of the effect size estimate. We aimed to develop a method to overcome these limitations.**

Methods: **We propose a procedure using generalized odds ratios to estimate the odds that a patient who received the investigational treatment will have a better outcome than a patient receiving standard treatment. This approach was validated against the permutation method using hypothetical clinical trial scenarios of neuroprotective effect, early recanalization effect, late recanalization effect, and random benefit.**

Results: **The generalized odds ratio approach had strong agreement with the permutation approach provided sample size was >15 patients per treatment arm. Simulation established that the confidence intervals generated were accurate. Ignoring patient pairs with tied mRS scores overestimates the treatment effect compared with splitting tied mRS scores.**

Conclusions: **In addition to all the advantages of the recently proposed permutation-based approach, our method generates confidence intervals without the need for intensive computational power. The resulting generalized odds ratios are particularly suitable for inclusion in meta-analyses and have a simple and intuitive connection with the number-needed-to-treat measure.**

Key words: clinical trial, epidemiology, outcome and process assessment, randomized controlled trials, statistical analysis, stroke

Introduction

The modified Rankin Scale (mRS) is the favored measure of functional outcome in phase 3 stroke trials (1). The mRS is a 7-point scale ranging from 0 (no symptoms) to 6 (death) (Table 1). Traditionally, the scale has been dichotomized as 0–1 versus 2–6 ('proportion with disability-free outcome') or 0–2 vs 3–6 ('proportion with independent outcome'). This is easy to understand but ignores shifts between individual levels of the mRS that may be clinically relevant.

Several methods of ordinal analysis of the full mRS have been proposed, with varying statistical properties (2–6). The Cochran–Mantel–Haenszel test (7) provides a *P*-value but not an associated treatment effect size measure. Proportional-odds logistic regression modeling (8) relies heavily on the assumption of proportionality of odds (also known as parallel regression assumption), which is often not satisfied in stroke trials (9).

A rank-based Mann–Whitney test provides a straightforward method for analysis of the mRS (10). Recently, Howard *et al.* (11) proposed a permutation-based approach for analysis of mRS that is closely related to the Mann–Whitney *U*-test (Wilcoxon rank-sum test). The test addresses the following question: 'If a patient is chosen at random from each treatment group and if they have different outcomes, what is the chance the patient who received the investigational treatment will have a better outcome than the patient receiving standard treatment?' This differs from the standard Wilcoxon–Mann–Whitney (WMW) approach in the treatment of tied observations. WMW assumes that half the tied scores had a lower mRS score for the patient assigned to the first treatment and half had a lower mRS score for the patient assigned to the second treatment. Howard *et al.* (11) argue that although the WMW approach is attractive analytically, including ties obscures the interpretation of the effect size. However, although it is assumption-free and highly clinically interpretable, this approach is computationally intensive; does not provide confidence intervals (CIs), which are an important indicator of study precision; and does not directly facilitate meta-analysis.

We aimed to develop and validate an assumption-free ordinal analysis of mRS using generalized odds ratios (GenOR) to provide an easily interpretable measure of effect size with CIs and number needed to treat (NNT), in addition to facilitating meta-analysis of studies.

Methods

We propose an analytical procedure that relies on GenOR (12). Instead of estimating probability per Howard *et al.'s* (11) approach, this procedure estimates the odds that for two patients chosen at random with one from each treatment group, the patient who received the investigational treatment will have a better outcome than the patient receiving standard treatment (illustrated in Fig. 1). The aim was to provide the additional benefits of confidence limits and *P*-values without being computationally demanding and to allow direct meta-analysis of studies

Correspondence: Leonid Churilov, Florey Institute of Neuroscience and Mental Health, University of Melbourne, Melbourne, Victoria, Australia.
E-mail: Leonid.Churilov@florey.edu.au

[1]Florey Institute of Neuroscience and Mental Health, University of Melbourne, Melbourne, Australia
[2]RMIT University, Melbourne, Australia
[3]Murdoch Childrens Research Institute, Melbourne, Australia
[4]Melbourne Brain Centre at the Royal Melbourne Hospital, Department of Medicine and Neurology, University of Melbourne, Melbourne, Australia

Received: 25 July 2014; Accepted: 29 July 2014; Published online 4 September 2014

Conflict of interest: None.

Funding: The research conducted at the Florey Institute is partially supported by the Operational Infrastructure Support Program of the State Government of Victoria.

DOI: 10.1111/ijs.12364

© 2014 World Stroke Organization

using the GenOR. A detailed description of the GenOR approach is provided in the online supplement.

Accuracy was validated by demonstrating that the GenOR provides both bias-free estimation of the true population GenOR and the correct coverage of 95% CIs and by comparing the performance of the GenOR with that of the permutation-based procedure of Howard *et al.* (11) under various treatment effect conditions using simulated data sets corresponding to four hypothetical clinical trial scenarios created by Saver and Gornbein (9):

Table 1 Modified Rankin Scale

Score	Description
0	No symptoms at all
1	No significant disability despite symptoms; able to perform all usual duties and activities
2	Slight disability; unable to perform all previous activities, but able to take care of self without assistance
3	Moderate disability; requiring some help, but able to walk without assistance
4	Moderately severe disability; unable to walk without assistance and unable to attend to own bodily needs without assistance
5	Severe disability; bedridden, incontinent, and requiring constant nursing care and attention
6	Dead

1. Neuroprotective: mild benefits experienced across all ranges of stroke severity
2. Early recanalization effect: substantial benefits across all ranges of stroke severity
3. Late recanalization effect: substantial benefits across wide range of stroke severity but with limited ability to achieve fully normal outcome
4. Random benefits: benefits clustered at unexpected health state transitions

The distributions were designed by Saver and Gornbein (9) to have 80% power at $\alpha = 0{\cdot}05$ for either WMW U-test or χ^2-test for two proportions, whichever had greatest power, at $n = 600$ per treatment arm. We created datasets that followed these treatment patterns and used them as source distributions for our simulation experiments (Fig. 2).

The agreement between the results produced by the analytical GenOR and the permutation method proposed by Howard *et al.* (11) was assessed using Lin's concordance coefficient (CC) (13) and further validated using reduced major axis regression (14). Reduced major axis regression allows separation of the disagreement into fixed and proportional components. If both methods were to produce identical readings, the corresponding scatter plot would fall onto the diagonal line of 'perfect concordance'. In cases when two methods provide readings that differ by a similar amount (fixed bias), the intercept of the reduced major axis

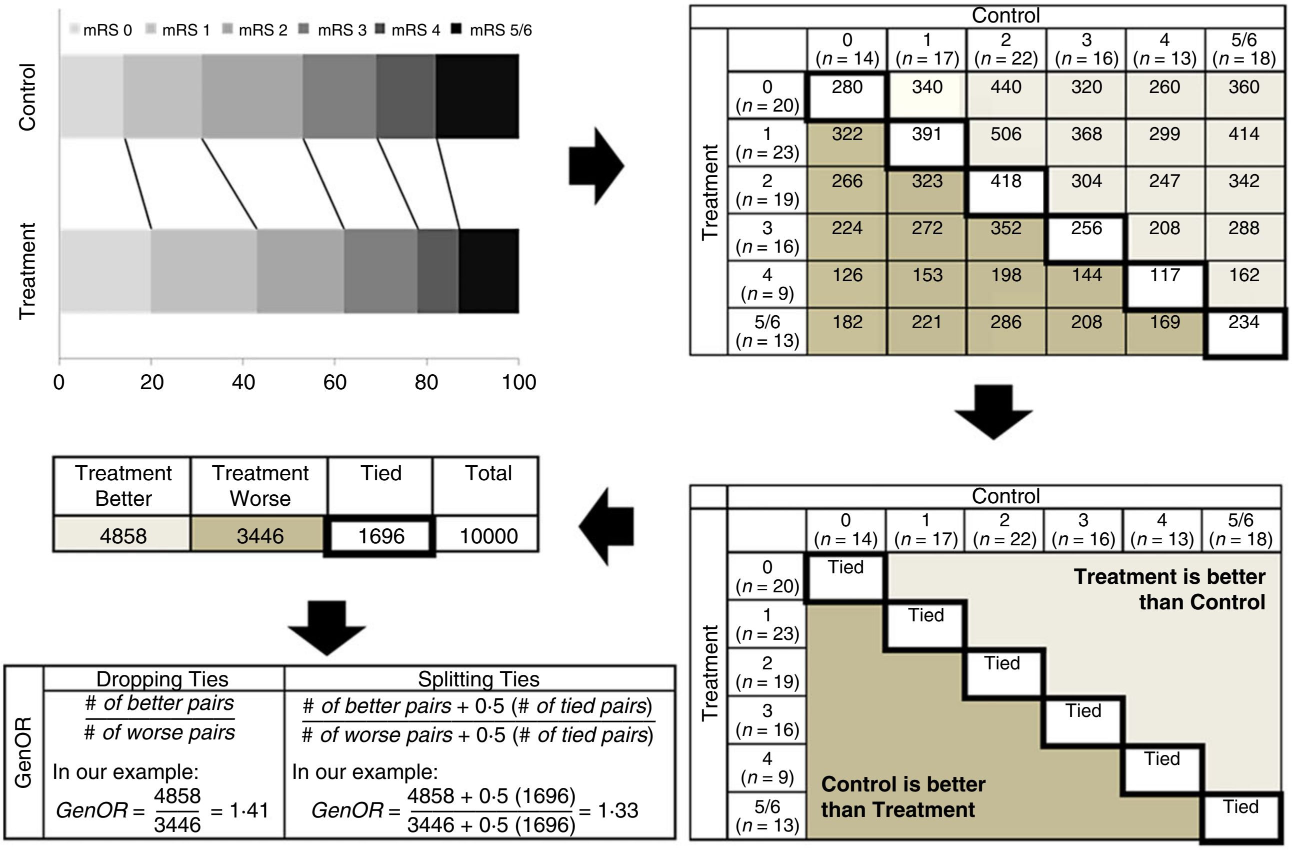

Fig. 1 Conceptual outline of generalized odds ratio (GenOR) approach using a hypothetical study with 100 subjects per arm. All pairs with better outcome in the treatment group are weighed against all pairs with worse outcome in the treatment group, and ties can be 'split' (in this case 50:50) or 'dropped' (excluded from consideration). Excluding ties inflates the estimated GenOR compared to splitting ties.

© 2014 World Stroke Organization

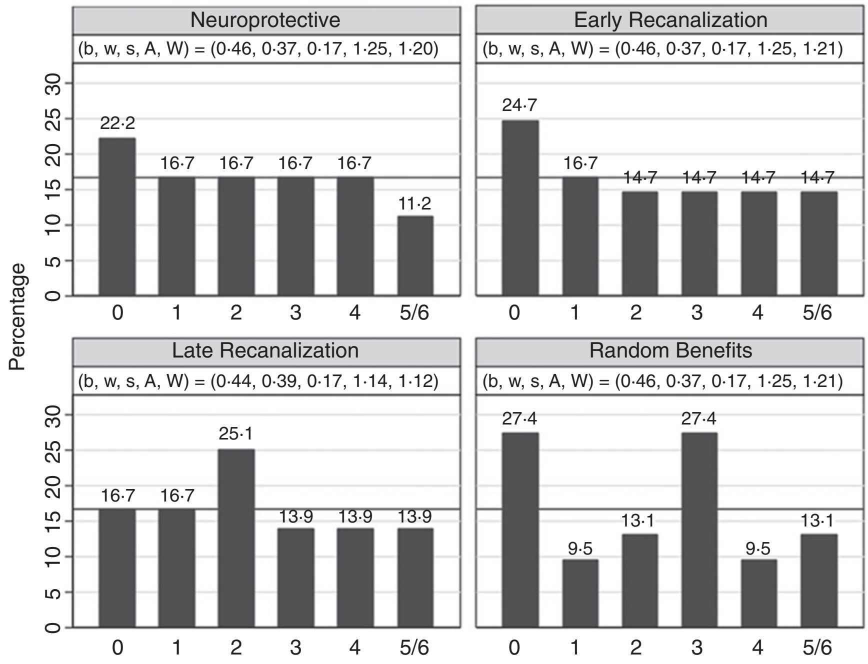

Fig. 2 Saver and Gornbein's (9) four hypothetical treatment effects. The solid horizontal line indicates the placebo group, with the bars in each modified Rankin Scale category representing the outcome in the treatment group. b/w/s, probability of treatment being better than/worse than/same as placebo; A, Agresti generalized odds ratio (GenOR); W, Wilcoxon–Mann–Whitney (WMW) GenOR. Note that the treatment of ties leads to the Agresti GenOR (ignores ties) always being greater than the WMW GenOR (50:50 splitting of ties).

regression line will differ from zero by that amount. If, on the other hand, the degree of disagreement changes with the magnitude of the readings, the slope of the reduced major axis regression line will differ from that of the line of perfect concordance, which is indicative of the presence of proportional bias.

The procedure for generating GenORs together with the corresponding 95% CIs was coded in Stata statistical software (v.13 IC, StataCorp, College Station, TX, USA). Two versions of GenOR are considered in the paper, as discussed in the Online Supplement: the original Agresti GenOR and the WMW GenOR. The difference between these two GenOR measures is in the way the tied observations are treated: while the Agresti GenOR ignores ('drops') the ties, the WMW GenOR splits the tied observations equally between the two treatment groups.

To investigate the ability of GenOR to produce an unbiased estimate of the true population GenOR, as well as corresponding 95% CIs that have correct coverage (i.e. truly contain the true population value in 95% of cases), we randomly generated 10 000 independent samples of $n = 600$ per treatment arm from each of the four hypothetical treatment effect distributions. The value 10 000 was chosen as it provided a sufficient number to return a normal sampling distribution of effect measures, while the sample size of 600 subjects per treatment arm was chosen based on power considerations.

To validate the performance of the analytical GenOR against that of the permutation method proposed by Howard *et al.* (11), the following series of simulations were performed:

1. Assessment of the agreement between the *P*-values generated by the analytical and permutation methods as a function of number of permutations: 50 independent samples of 600 patients per treatment arm from each of the four hypothetical treatment effect distributions for the scenarios with 10, 100, 1000, and 10 000 permutations required by Howard's method were generated, and Lin's CCs for each permutation scenario were calculated. The number of permutations was specifically experimented with in order to investigate how many permutations in Howard's method would be required to achieve convergence between the permutation and the analytical results.
2. Assessment of the agreement between the *P*-values generated by the analytical and permutation methods (30 000 permutations per independent sample) as a function of sample size: 200 independent samples from each of the four hypothetical treatment effect distributions were generated for sample sizes of 5, 10, 15, 25, 38, 50, and 100 subjects per treatment arm, and Lin's CC as well as reduced major axis regression slope and intercept were calculated for each sample size value.
3. Assessment of the agreement for stratified analysis between analytical and permutation methods (5 000 permutations per independent sample): 50 independent samples from each of the four hypothetical treatment effect distributions were generated for a sufficiently large sample size of 100 subjects per treatment arm, and Lin's CC as well as reduced major axis regression slope and intercept were calculated.

Finally, to estimate the degree of the disagreement between the Agresti GenOR ('ignoring/dropping ties' strategy) and WMW GenOR ('splitting ties' strategy) as a function of the sample size (values: 5, 10, 15, 25, 38, 50, and 100 subjects per treatment arm), 200 independent samples from each of the four hypothetical treatment effect distributions were generated for each sample size,

© 2014 World Stroke Organization

and respective estimates of reduced major axis regression slope and intercept were obtained.

The GenOR and permutation methods were then illustrated using mRS data from the National Institute of Neurological Disorders and Stroke tissue plasminogen activator (NINDS tPA) trial (15).

Results

Unbiased estimates of population GenOR with correct 95% confidence interval coverage

The four hypothetical treatment effect distributions are illustrated in Fig. 2. The horizontal line indicates the placebo group. Corresponding text shows the probabilities that a randomly selected treatment patient would have a better, worse, or equal mRS score compared with a randomly selected placebo patient, along with the corresponding Agresti and WMW GenORs. The WMW GenOR is consistently lower than the Agresti GenOR because of the splitting of ties that are ignored using the Agresti (or Howard *et al.*) method. For example, in the early recanalization model, the probability that the treatment patient had a better outcome than the placebo patient was 46%, the probability they had a worse outcome was 37%, and the probability that both were equal was 17%. When ties are ignored, this leads to a GenOR of 1·25, meaning a treatment patient is 1·25 times more likely to have a better rather than worse outcome when compared with a randomly chosen placebo patient. When pairs with tied outcomes are split evenly between better and worse outcome, the treatment patient was 1·21 times more likely to have a better rather than worse outcome when compared with a randomly chosen placebo patient.

Over 10 000 simulated trials with 600 subjects in each treatment arm, Agresti GenOR and WMW GenOR, produced unbiased estimates of the respective true odds ratios, and the 95% CI included the true values of the treatment effect in 95% of simulated trials for all treatment scenarios, indicating correct coverage. Therefore both Agresti GenOR and WMW GenOR provided a reliable method of estimating the respective true odds of improvement for patients receiving treatment.

Agreement between the permutation-based and analytical approaches as a function of number of permutations

The agreement between the *P*-values obtained by permutation versus the analytical GenOR method as a function of the number of permutations is shown in Fig. 3A and B. For all treatment effect scenarios, as the number of permutations increased, the permutation *P*-value converged on the analytical result, and by 1 000 permutations there was near-perfect concordance. There was no appreciable difference in the concordance for Agresti GenOR and WMW GenOR. There also appears to be little difference between the treatment effect models in the number of permutations required for the *P*-values to converge. Very similar *P*-values were obtained by performing 10 000 and 100 000 permutations, thus demonstrating that the *P*-values for the asymptotic solution and the permutation method converge between 1000 and 10 000 permutations.

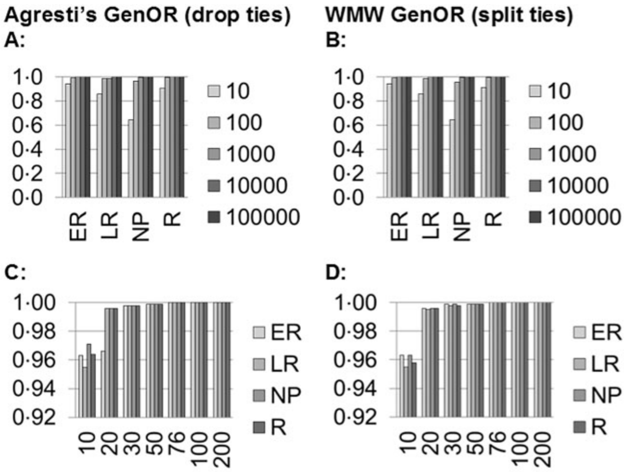

Fig. 3 The agreement between *P*-values calculated by permutation method and using Agresti (drop ties) and Wilcoxon–Mann–Whitney (WMW; split ties) generalized odds ratios (GenORs). Lin's concordance coefficient is shown as a function of number of permutations (a,b) and total sample size (c,d). Treatment patterns: ER, early recanalization; LR, late recanalization, NP, neuroprotection, R, random benefit.With ≥1000 permutations the *P*-value converges with that calculated using Agresti/WMW GenOR. With sample size ≥15 patients per group, the *P*-value for Agresti/WMW GenOR converges with the permutation *P*-value.

Agreement between the permutation-based and analytical approaches as a function of sample size

Figure 3C and D and Table S1 (online) demonstrate the analogous comparison as a function of the sample size. For all treatment effect scenarios, the agreement between analytical and permutation methods increased as the sample size increased. The magnitude of Lin's CC remained above 0·95 for all the sample sizes, signifying excellent agreement according to Landis and Koch (16). The agreement became practically indistinguishable from 1 for sample sizes of 15 or more subjects per arm, irrespective of the treatment scenario used. This is consistent with the total sample size of 30 being traditionally regarded as a quasi-threshold for using normal-distribution-based approximations.

Comparison of adjustment for baseline variables between the stratified permutation-based approach and the stratified Agresti/WMW GenOR

Excellent agreement between the stratified permutation-based and analytical approaches was observed for all treatment scenarios, as manifested by the values of Lin's CC above 0·99 (Table 2). The reduced major axis slope being very close to 1 and intercept values very close to 0 indicated that neither method consistently produced higher/lower outcomes and that the magnitude of the outcome produced did not affect the degree of agreement between the methods.

Ignoring tied observations consistently overestimates treatment effect compared to splitting tied observations

Irrespective of the treatment pattern, ignoring ('dropping') ties when calculating effect sizes consistently overestimated the effects compared with 'splitting' the ties. As detailed in Table S2 (online), this comparative overestimation was higher for smaller samples,

© 2014 World Stroke Organization

Table 2 Agreement between *P*-values generated by stratified permutation approach and stratified generalized odds ratio approach

Treatment pattern	Stratified Agresti generalized odds ratio (split ties)	Stratified Wilcoxon–Mann–Whitney generalized odds ratio (drop ties)
Neuroprotective		
Lin's concordance coefficient	0·997	0·998
95% confidence interval	0·995, 0·999	0·998, 0·999
Slope (intercept)*	1·024 (–0·007)	1·015 (–0·009)
Early recanalization		
Lin's concordance coefficient	0·998	0·999
95% confidence interval	0·998, 0·999	0·998, 1·000
Slope (intercept)*	1·016 (–0·011)	0·993 (–0·003)
Late recanalization		
Lin's concordance coefficient	0·999	0·998
95% confidence interval	0·999, 1·000	0·997, 0·999
Slope (intercept)*	0·993 (0·001)	1·003 (–0·005)
Random benefits		
Lin's concordance coefficient	0·999	0·998
95% confidence interval	0·998, 0·999	0·998, 0·999
Slope (intercept)*	1·009 (–0·006)	1·007 (–0·005)

Stratified permutation approach follows Howard *et al*. (11). Data in every cell are based on 50 independent samples, each containing 100 subjects per treatment arm, and on 5000 permutations per independent sample.
*Slope and intercept generated by reduced major axis regression; note that a slope of 1 and intercept and 0 would mean the absence of proportional and fixed bias, respectively.

but even for the relatively large sample size of 200 subjects per group, a considerable proportional bias between the two methods (reduced major axis regression slope around 0·85) was obvious.

Performance in the NINDS tPA trial data

Ninety-day mRS data are freely available for the NINDS tPA trial (15). For this analysis, mRS 5 and 6 were combined to maintain consistency with Howard *et al*. In 49% of possible pairs of treatment and placebo patients, the treatment patient had a better outcome than the placebo patient. In 33% of pairs, the placebo patient had the better outcome, and in 18% of pairs the mRS was tied. This leads to the Agresti GenOR (1·44, 95% CI: 1·16 to 1·8, $P = 0{\cdot}00078$; ordinal NNT = 5·63, 95% CI: 3·59 to 13·85), and when pairs of patients who have the same mRS are not ignored, there is a decrease in effect size, and the respective estimate of WMW GenOR is 1·35 (95% CI: 1·12 to 1·61, $P = 0{\cdot}00094$; ordinal NNT = 6·65, 95% CI: 4·21 to 16·39). Note that because ties are ignored in the Agresti GenOR, and consequently the sample size is reduced, the 95% CIs are slightly wider than for WMW GenOR. The ordinal NNTs resulting from WMW GenOR approaches are consistent with the unmatched ordinal NNTs reported by Bath *et al*. (6) for NINDS data as 6·65 (95% CI: 3·98, 20·1).

For illustrative purposes, if adjustment for baseline characteristics is required and patients are stratified by age (above versus below 70 years old) and stroke severity (mild: National Institutes of Health Stroke Scale [NIHSS] 7 or below; moderate: NIHSS 8 to 15 inclusive; severe: NIHSS 16 or above), the resulting Agresti GenOR equals 1·43 (95% CI: 1·13, 1·8, $P = 0{\cdot}0014$; ordinal NNT = 5·68, 95% CI: 3·48 to 16·65) and WMW GenOR equals 1·29 (95% CI: 1·08, 1·55, $P = 0{\cdot}0052$; ordinal NNT = 7·8, 95% CI: 4·66 to 25·06). Applying a proportional odds 'shift analysis' approach (8) implemented as an ordinal logistic regression model with the treatment group, age, and NIHSS as inputs results in an adjusted proportional OR of 1·57 (95% CI: 1·17 to 2·09, $P = 0{\cdot}002$). Importantly, a fundamental 'parallel regression/proportional odds' assumption underlying the proportional odds approach to ordinal analysis was violated for the age variable (Brant test $P = 0{\cdot}031$), casting doubt on the validity of the proportional odds model for NINDS data.

Ease of meta-analysis

GenOR behaves just like odds ratios and hazard ratios in the sense that the sampling distribution of log(GenOR) follows an approximately normal distribution (12). Therefore, GenOR and its CI can be compared with those obtained from any other ordinal responses from other studies and easily included in a meta-analysis. Figure 4 presents a forest plot illustrating a fictitious meta-analysis of 10 randomly generated hypothetical studies with significant heterogeneity of treatment effect and the overall GenOR of 1.

Discussion

This method provides accurate and easily interpretable ordinal analysis of the mRS. It provides a *P*-value, CI and NNT and could be applied to any ordinal scale. A particularly useful property of GenOR is the ability to directly meta-analyze data from different trials.

The WMW rank-sum test is commonly used to test the null hypothesis of no treatment effect when the outcome data are not parametrically distributed. The null hypothesis that WMW GenOR = 1 is a simple rearrangement of the null hypothesis in the WMW *U*-test. Therefore, both tests produce the same *P*-value. Agresti GenOR is similar to WMW GenOR and the WMW *U*-test, except that tied values are ignored. In the four treatment effect scenarios, the permutation *P*-values converged to the values

© 2014 World Stroke Organization

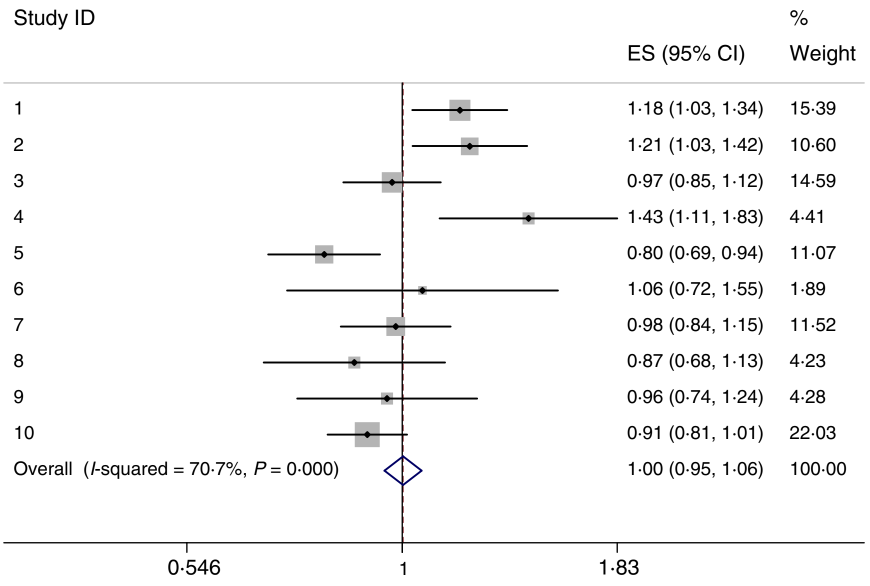

Fig. 4 Forest plot illustrating a fictitious meta-analysis of the generalized odds ratio (GenOR) effects resulting from 10 randomly generated hypothetical studies with significant heterogeneity of treatment effect and the overall GenOR of 1.

found by the analytic solution given by Agresti (12) and later by O'Brien (17). The two-sided *P*-value for the WMW GenOR calculated via permutation and analytic solution also converged.

As the analytic solution allows for the estimation of standard errors, it is possible to construct a CI for the effect measure. In contrast, the permutation method provides only a *P*-value for the test of the null hypothesis.

In all cases, the WMW GenOR is more conservative than the Agresti GenOR. This is because the ties are split evenly between the denominator and numerator of the odds ratio, bringing the ratio closer to 1. There are other ways in which ties could be handled; for example, instead of an even split they could be placed with the pairs where the treatment patient had a better outcome than the control patient. The odds ratio then estimates how many times more likely a randomly chosen treatment patient is to have an outcome no worse than a randomly chosen control patient than he or she is to have a worse outcome. However the ties are handled, ignoring them leads to difficulties in interpreting the odds ratio when a large proportion of data are ignored, and the reduced sample size reduces the precision of the estimate. If, for example, GenOR = 2 but this ignored 50% of the observations that were tied, a more complete and honest statement would be 'You are twice as likely to have a better outcome than a worse outcome with treatment, but there's also a 50% chance you would have the same outcome as you would without treatment.'

A limitation of this method and that of Howard *et al.* (11) is that adjustment for continuous variables (e.g., age) is not directly possible. The suggested solution to this is to use the stratified WMW GenOR, which allows adjustment for categorical confounders. Continuous variables such as age and baseline stroke severity could be grouped in predefined categories, and then a stratified version of the WMW GenOR could be used.

Adopting either Howard *et al.*'s (11) approach or the approach proposed in this paper also provides an easily understandable and natural link to the concept of the ordinal NNT discussed recently by Bath *et al.* (2) for unmatched comparison of all subjects, where the NNT is derived as the reciprocal of the difference between 'proportion better' and 'proportion worse'.

An important advantage of the odds-based approach as compared with the probabilities-based approach proposed by Howard *et al.* (11) is that GenOR provides a simple effect size that can be compared with similar outcomes from ordinal responses (including continuous ones) from other studies (17). This opens up an attractive opportunity for their use in meta-analysis, as if they were odds ratios generated from studies with binary outcomes, as well as the possibility of consistent visual representation by means of forest plots. In addition, as log(GenOR) follows an approximately normal distribution, GenOR can be readily used as an outcome for dose–response studies and incorporated into adaptive designs.

In conclusion, both Agresti and WMW GenORs provide a natural extension to the WMW test by presenting an interpretable, consistent effect measure that requires no assumptions about the distribution of ordinal outcome data and provides adequate facilities for stratified analysis. Given the ability to calculate a CI and reduced computational intensity, we feel they should be preferred to the permutation method as long as the sample size is no less than 15 subjects per treatment arm. Splitting the ties as in the WMW GenOR approach is favored, as it provides more realistic effect size estimates with tighter CIs as compared with the Agresti GenOR.

References

1 Lees KR, Bath PMW, Schellinger PD *et al.* Contemporary outcome measures in acute stroke research: choice of primary outcome measure. *Stroke* 2012; **43:**1163–70.
2 Bath PMW, Lees KR, Schellinger PD *et al.* Statistical analysis of the primary outcome in acute stroke trials. *Stroke* 2012; **43:**1171–8.
3 Optimizing the Analysis of Stroke Trials (OAST) Collaboration. Can we improve the statistical analysis of stroke trials? Statistical reanalysis of functional outcomes in stroke trials. *Stroke* 2007; **38:**1911–5.

© 2014 World Stroke Organization

4 Optimizing the Analysis of Stroke Trials (OAST) Collaboration. Calculation of sample size for stroke trials assessing functional outcome: comparison of binary and ordinal approaches. *Int J Stroke* 2008; **3:**78–84.
5 Optimizing the Analysis of Stroke Trials (OAST) Collaboration. Should stroke trials adjust functional outcome for baseline prognostic factors? *Stroke* 2009; **40:**888–94.
6 Bath PMW, Hogg C, Tracy M, Pocock S, on behalf of OAST Collaboration. Calculation of numbers-needed-to-treat in parallel group trials assessing ordinal outcomes: case examples from acute stroke and stroke prevention. *Int. J. Stroke* 2011; **6:**472–80.
7 Lees KR, Zivin JA, Ashwood T *et al.* NXY-059 for acute ischemic stroke. *N Engl J Med* 2006; **354:**588–600.
8 Saver JL. Novel end point analytic techniques and interpreting shifts across the entire range of outcome scales in acute stroke trials. *Stroke* 2007; **38:**3055–62.
9 Saver JL, Gornbein J. Treatment effects for which shift or binary analyses are advantageous in acute stroke trials. *Neurology* 2009; **72:**1310–5.
10 Koziol JA, Feng AC. On the analysis and interpretation of outcome measures in stroke clinical trials: lessons from the SAINT I Study of NXY-059 for acute ischemic stroke. *Stroke* 2006; **37:**2644–7.
11 Howard G, Waller JL, Voeks JH *et al.* A simple, assumption-free, and clinically interpretable approach for analysis of modified Rankin outcomes. *Stroke* 2012; **43:**664–9.
12 Agresti A. Generalized odds ratios for ordinal data. *Biometrics* 1980; **36:**59–67.
13 Lin L. A concordance correlation coefficient to evaluate reproducibility. *Biometrics* 1989; **45:**255–68.
14 Ludbrook J. Linear regression analysis for comparing two measurers or methods of measurement: but which regression? *Clin Exp Pharmacol Physiol* 2010; **37:**692–9.
15 National Institute of Neurological Disorders and Stroke rt-PA Stroke Study Group. Tissue plasminogen activator for acute ischemic stroke. *N Engl J Med* 1995; **333:**1581–7.
16 Landis JR, Koch GG. The measurement of observer agreement for categorical data. *Biometrics* 1977; **33:**159–74.
17 O'Brien RG, Castelloe J. Exploiting the link between the Wilcoxon–Mann–Whitney test and a simple odds statistic; in Proceedings of the Thirty-first Annual SAS Users Group International Conference. Cary, NC, SAS Institute Inc., 2006:209–31.

Supporting information

Additional Supporting Information may be found in the online version of this article at the publisher's web-site:

An improved method for simple, assumption-free ordinal analysis of the modified Rankin scale using generalized odds ratios.

© 2014 World Stroke Organization
